# Inactivation of Bacteria Using Bioactive Nanoparticles and Alternating Magnetic Fields

**DOI:** 10.3390/nano11020342

**Published:** 2021-01-29

**Authors:** Vitalij Novickij, Ramunė Stanevičienė, Rūta Gruškienė, Kazimieras Badokas, Juliana Lukša, Jolanta Sereikaitė, Kęstutis Mažeika, Nikolaj Višniakov, Jurij Novickij, Elena Servienė

**Affiliations:** 1 Faculty of Electronics, Vilnius Gediminas Technical University, 03227 Vilnius, Lithuania; jurij.novickij@vgtu.lt; 2 Laboratory of Genetics, Nature Research Centre, 08412 Vilnius, Lithuania; ramune.staneviciene@gamtc.lt (R.S.); juliana.luksa@gamtc.lt (J.L.); 3 Faculty of Fundamental Sciences, Vilnius Gediminas Technical University, 10223 Vilnius, Lithuania; ruta.gruskiene@vgtu.lt (R.G.); jolanta.sereikaite@vgtu.lt (J.S.); 4 Institute of Photonics and Nanotechnology, Vilnius University, 10257 Vilnius, Lithuania; kazimieras.badokas@ff.vu.lt; 5 Center for Physical Sciences and Technology, 02300 Vilnius, Lithuania; kestutis.mazeika@ftmc.lt; 6 Faculty of Mechanics, Vilnius Gediminas Technical University, 03224 Vilnius, Lithuania; nikolaj.visniakov@vgtu.lt

**Keywords:** electromagnetic fields, food processing, sterilization, nisin, *L. innocua*

## Abstract

Foodborne pathogens are frequently associated with risks and outbreaks of many diseases; therefore, food safety and processing remain a priority to control and minimize these risks. In this work, nisin-loaded magnetic nanoparticles were used and activated by alternating 10 and 125 mT (peak to peak) magnetic fields (AMFs) for biocontrol of bacteria *Listeria innocua,* a suitable model to study the inactivation of common foodborne pathogen *L. monocytogenes.* It was shown that *L. innocua* features high resistance to nisin-based bioactive nanoparticles, however, application of AMFs (15 and 30 min exposure) significantly potentiates the treatment resulting in considerable log reduction of viable cells. The morphological changes and the resulting cellular damage, which was induced by the synergistic treatment, was confirmed using scanning electron microscopy. The thermal effects were also estimated in the study. The results are useful for the development of new methods for treatment of the drug-resistant foodborne pathogens to minimize the risks of invasive infections. The proposed methodology is a contactless alternative to the currently established pulsed-electric field-based treatment in food processing.

## 1. Introduction

Foodborne diseases associated with bacteria represent a serious health problem, which may be fatal in some cases [1,2]. As a result, significant efforts are made to ensure food safety and adequate management of the bacterial contaminants. Thus, processing may involve thermal sterilization [3,4], pulsed electric field treatment [5], cold-plasma treatment [6], natural bacteriocins [7,8] or nanotechnological methods [9,10]. The synergistic approaches when several different methodologies are combined frequently deliver promising results in food control [11,12,13]. 

Many of the methodologies rely on the chemical interactions between the bacteria and the target molecule, which sooner or later may result in occurrence of the treatment-resistant microorganisms [14,15,16]. It is especially true in the context of antibiotics and bacteriocins, when bacteria through modifications of their cell envelope (i.e., charge and thickness) can develop resistance to the chemical treatment [17,18]. The pharmaceutical industry and health care systems have been combating antibiotic-resistant strains of bacteria for more than 60 years [19], and nisin is currently the most popular bacteriocin used in the food sector [20]. It is efficient against many Gram-positive bacteria, while still being unstable in alkaline environment or during exposure to special protease [21,22]. Therefore, nisin nanoencapsulation is one of the best validated methods in order to prolong its bactericidal time, improve storage performance or obtain a sustained release [22,23,24]. Nevertheless, nanoencapsulation does not solve all the problems, and nisin still remains ineffective against Gram-negative bacteria or bacteria with thick cytoplasmic membrane and cell wall [25,26,27]. As a result, other food processing methods such as hydrostatic pressure techniques [28], cold plasma [29], ultraviolet light [30], ultrasound [31] or pulsed electric fields [32] have been extensively focused on for their use in the improvement of food safety. Nevertheless, the best effects are expected when a synergism between various methods is acquired [33]. Therefore, in order to improve the efficacy of nisin and induce a synergistic response, methods affecting the cell wall and plasma membrane are required. 

One of the solutions is to use physical methods for cell permeabilization and thus allow nisin to incorporate itself in the bacterial cell membrane by binding to essential precursors for cell wall biosynthesis, which ultimately leads to formation of pores, loss of solutes in bacteria and subsequent cell death [34]. In our previous works we have successfully shown that the efficacy of nisin nanoparticles can be effectively improved by electroporation [35,36]. Combination with mild thermal treatment further improves the efficacy even against Gram-negative bacteria in stationary growth phase [37]. However, application of pulsed electric fields (PEFs) involves metal electrodes being in direct contact with the treated sample, which results in ion release, electrode degradation due to electrolysis, various electrochemical reactions, pH gradients or voltage breakdowns [38,39,40,41,42]. All these factors to a certain extent can affect the quality of food and are considered as a limitation of PEF-based techniques. 

One of the solutions to overcome mentioned above limitations could be the application of magnetic fields, which do not require a direct contact with the sample. The phenomenon of contactless electroporation using pulsed magnetic fields and conductive nanoparticles has been confirmed recently [43]. However, in such a case, high power setups are required, and the methodology is still far from the capability to scale it industrially. Therefore, application of magnetic nanomaterial can be introduced to achieve a profound inactivation effect in low amplitude, but high frequency magnetic fields [44] enabling a multi-factorial treatment including magnetic hyperthermia. The parametrical flexibility and efficient removability of the nanoparticles from food can be highlighted as the main advantages of such methodology [45]. In order to further improve the efficacy, the magnetic nanoparticles can be functionalized and serve as drug carriers for targeted treatment [46]. Previously we tried to develop nisin-functionalized magnetic nanoparticles [47] for controlled release using high frequency alternating magnetic fields (AMFs) [48], but it was shown that the 10 mT, 100 kHz magnetic field is ineffective for potentiation of nisin-based treatment when short exposure times are used (2 min). Therefore, in this work, we employed a more powerful 125 mT, 200 kHz magnetic setup and increased the exposure times. The 10 mT, 100 kHz setup was used as a reference, and equivalent parametric protocols (exposure time-wise) were derived. 

The results are useful for the development of new methods for treatment of the drug-resistant foodborne pathogens to minimize the risks of invasive infections. We show that it is not solely the thermal stress influencing bioactivity of nisin nanoparticles, but also the alternating magnetic fields significantly improve the efficacy. The actuality of the proposed methodology lies in the contactless nature of the treatment, which is advantageous in terms of contamination and/or electrochemical reactions, which are typical for widely-implemented PEF-based treatment chambers.

## 2. Materials and Methods

### 2.1. Alternating Magnetic Fields

The experimental setup consisted of two generators: (1) a low magnetic field (LMF) 10 mT, 100 kHz generator and (2) a high magnetic field (HMF) 125 mT, 200 kHz generator. The coil of the LMF generator was made from hollow (5 mm diameter) copper wire resulting in a 1 layer 8 winding solenoid structure (inner effective diameter of 23 mm). The coil of the HMF generator was made from hollow (8 mm diameter) copper wire resulting in a 1 layer 3 winding solenoid structure (inner effective diameter of 10 mm). Liquid cooling was used to prevent heating of the coils. Both generators were compatible with 0.2 mL polymerase chain reaction (PCR) sterile tubes (Quali Electronics Inc., Columbia, SC, USA). 

**Figure 1:**
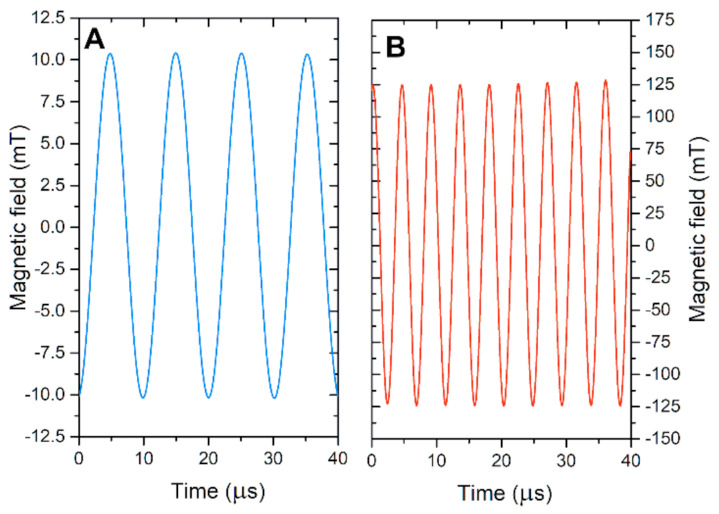
Measured alternating magnetic field waveforms. (**A**) 100 kHz low magnetic field generator and (**B**) 200 kHz high magnetic field generator.

The measured waveforms of both AMF generators are presented in Figure 1. The waveforms were acquired using a calibrated loop sensor (VGTU, Vilnius, Lithuania) and post-processed in OriginPro 8.5 Software (OriginLab, Northampton, MA, USA).

The exposure time was controlled to establish a dose-dependent cellular response. For the LMF generator the 5, 30 and 60 min protocols were employed, while for the HMF the 5, 15 and 30 min protocols were used. 

### 2.2. Thermal Influence

The thermal influence was estimated using a compact Pt1000 sensor (Innovative Sensor Technology, Wattwil, Switzerland). Temperature was measured with a varied time step (5–30 min) to grasp the moment of temperature saturation. In order to prevent the influence of eddy currents on the sensor response during AMF pulses, the pulse sequences were stopped for several seconds to acquire the measurement. After that, the pulsing was resumed for a further time step. For the LMF protocols the temperature did not exceed 37 °C, while for HMF it was below 45 °C. In order to estimate the influence of thermal stress and to allow adequate evaluation of the magnetic field-mediated methodology, a separate experiment where the bacterial cells were incubated in 37 and 45 °C for 5, 15, 30 and 60 min was performed. The experimental scheme included cells separately and with nisin, nisin nanoparticles (CaN) and nisin-free nanoparticles (CaO) to mirror the experiment with magnetic fields. 

### 2.3. Nisin-Loaded Magnetic Nanoparticles

Nisin-loaded iron oxide magnetic nanoparticles (NPs) were prepared as previously described [47]. Briefly, under vigorous stirring, 0.587 g FeCl_3_·6H_2_O and 0.278 g FeSO_4_·7H_2_O were mixed in 10 mL of water and heated to 80 °C under nitrogen in a three-necked flask. Then, 3.5 mL of NH_4_OH (10%) was dropped into the solution. After reaction for 30 min at 80 °C, 0.3 g of calcium citrate in 0.6 mL water was added directly into the reaction solution. The temperature was increased to 95 °C, and stirring continued for an additional 90 min. Then, the solution was cooled down to room temperature naturally. The iron oxide particles were separated by a magnet from reaction mixture, washed with deionized water several times and dried at 45 °C for 12 h. The prepared dried powder was stored in the refrigerator. Before use, the required amount of iron oxide was re-dissolved in water using an ultrasonic water bath for 3 h and centrifuged at 6400× *g* for 2 h. The final iron oxide nanoparticle solution was used for the following nisin loading. Synthesized iron oxide nanoparticles capped with citric acid corresponded to Fe_2_O_3_ phase (Maghemite-C, ICDD Card No. 00-039-1346) with a possible small quantity of magnetite as judged with the X-ray diffraction method. 

A vibrating sample magnetometer was used for the magnetization measurement of the sample. The lock-in amplifier SR510 (Stanford Research Systems, Sunnyvale, CA, USA) was applied for the detection of the signal from the sense coils generated by vibrating sample. A Gauss/Teslameter FH-54 (Magnet Physics Dr. Steingroever GmbH, Cologne, Germany) was used to measure the magnetic field strength between the poles of the laboratory magnet, which was supplied by the power source SM 330-AR-22 (Delta Elektronika, ND Zierikzee, Netherlands). The Mössbauer spectrum was measured using the ^57^Co (Rh) source in the transmission geometry with a Mössbauer spectrometer (Wissenschaftliche Elektronik GmbH, Starnberg, Germany). The Mössbauer spectrum was fitted to hyperfine field distributions applying WinNormos software (version 3.0 by R.A.Brand, Wissenschaftliche Elektronik GmbH, Starnberg, Germany). 

**Figure 2:**
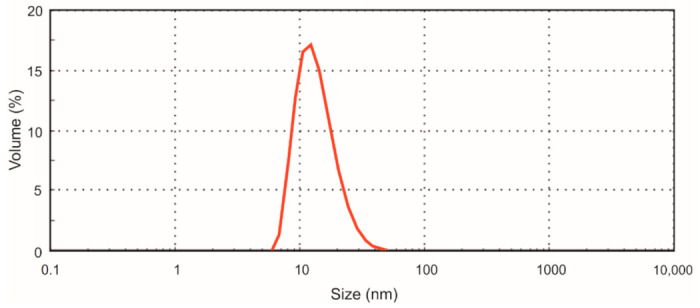
Size distribution of nisin-loaded iron oxide magnetic nanoparticles.

For the preparation of nisin-loaded particles, a nisin solution in water at the concentration of 10 μg/mL was added dropwise to the iron oxide nanoparticle solution (0.05 mg/mL) at the ratio 1/4 (*v/v*) under constant stirring at room temperature. The final concentration of nisin in the product was 2 μg/mL, and that of iron oxide nanoparticles in the solution was 0.04 mg/mL. For the preparation of control, instead of nisin solution, water was used. The solution of prepared nisin-loaded NPs was stored at +4 °C. Nisin loading on the particles was confirmed by Fourier transform infrared spectroscopy and thermogravimetric analysis. The diameter of nisin-loaded particles determined by atomic force microscopy and the hydrodynamic diameter determined by dynamic light scattering method was 11.3 ± 1.4 and 26.4 ± 2.0 nm, respectively [44]. The size distribution (hydrodynamic radius) of nisin-loaded particles is presented in Figure 2. 

### 2.4. Bacterial Cells

The bacteria *Listeria innocua* CECT 910T (kindly provided by Maria Joao Fraqueza, University of Lisbon, Lisbon, Portugal) was cultivated in brain heart infusion (BHI) medium (1.25% brain extract, 0.5% heart extract, 1% peptone, 0.2% dextrose, 0.5% NaCl, 0.25% Na_2_HPO_4_) for 16–18 h with continuous shaking at 37 °C. Overnight grown cells were collected by centrifugation at 6000× *g* for 5 min, washed 3 times with 1 mol/L sorbitol, re-suspended in 1 mol/L sorbitol at a final concentration of about 1 × 10^9^ cells/mL and used in AMF treatment experiments.

For the analysis of bacteria viability, the cells of *L. innocua* in 1 mol/L sorbitol (50 μL) were mixed with equal volume of nisin-unloaded/loaded magnetic nanoparticle solution (*v*/*v* 1:1) and treated with AMFs. The final concentration of the nisin in the mixture was 1 μg/mL, and the concentration of iron oxide nanoparticles was −0.02 mg/mL. After the treatment (5–60 min), the samples were incubated at room temperature (20 °C) for total time of (treatment time + incubation = 1 h, e.g., 5 min AMF treatment + 55 min incubation) without agitation, serial dilutions were performed in sterile 0.9% NaCl and 50 μL of each solution was spread onto BHI-agar plates with incubation following overnight at 37 °C. After the incubation, colonies were counted as colony forming units (CFU), and the mean value of CFU/mL was calculated. In subsequent bacteria viability experiments, the volume of nisin-unloaded/loaded magnetic nanoparticles was doubled (*v*/*v* 2:1) and tripled (*v*/*v* 3:1). As a reference, nisin-only solution was also implemented in the study.

### 2.5. Scanning Electron Microscopy

For the preparation of scanning electron microscopy (SEM), *L. innocua* cells (1 × 10^9^ CFU/mL) were incubated with nanoparticles and/or treated by AMFs and followed serial dilutions in sterile water. Then, 5 μL measurements of solutions were dropped onto the specimen stubs covered with copper foil tape and gently dried at room temperature. For the microscopy preparation, samples were sputter coated with a 25 nm gold layer using a Q150T ES sputter coater (Quorum Technologies, Laughton, UK). Twenty or more images per cell treatment were obtained using an Apollo 300 (CamScan, Cambridge, UK) scanning electron microscope operating at 15 kV. 

### 2.6. Statistical Analysis

One-way analysis of variance (ANOVA; *p* < 0.05) was used to compare results. If ANOVA indicated a statistically significant result (*p* < 0.05), Tukey’s honest significance difference (HSD) multiple comparison test for evaluation of the difference was used. The data were post-processed in OriginPro software (OriginLab, Northhampton, MA, USA). All experiments were performed with at least three repetitions, and the treatment efficiency is expressed as mean ± standard deviation.

## 3. Results

**Figure 3:**
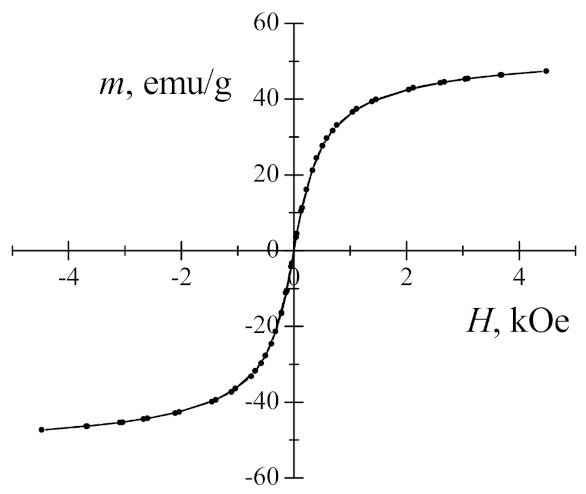
Magnetization dependence of maghemite/magnetite nanoparticles on the applied magnetic field.

Before the experiments with cells, Mössbauer spectroscopy and magnetization measurements of iron oxide magnetic nanoparticles were performed. As shown in Figure 3, the magnetization dependence has no hysteresis or remnant magnetization and thus is characteristic of superparamagnetic nanoparticles. The saturation magnetization of maghemite/magnetite nanoparticles of ≈48 emu/g is lower in comparison with 92 emu/g of bulk magnetite. For nanosized materials, saturation magnetization decreases because of the magnetically dead layer, magnetic disorder of the surface layer and the presence of nonmagnetic adsorption on surface materials. 

**Figure 4:**
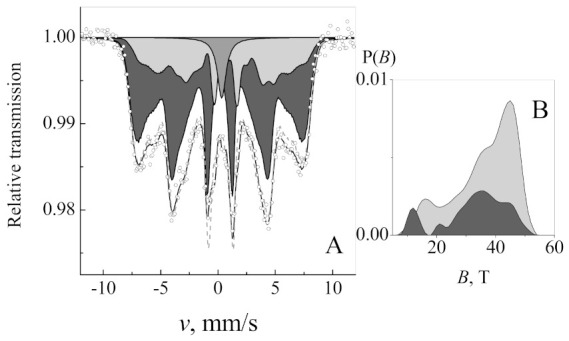
Mössbauer spectrum (**A**) of maghemite/magnetite nanoparticles at 21 °C temperature and two hyperfine field distributions P(*B*) (**B**) applied for fitting. Dashed gray line (**A**) given for comparison indicates fitting using one hyperfine field distribution.

The broadening of spectral lines of the Mössbauer spectrum (Figure 4) showed superparamagnetism of nanoparticles. Two hyperfine field distributions were used to fit to the spectrum (Table 1). The two hyperfine field distributions P(*B*), which differ by isomer shift, give better quality of fitting than only one distribution as shown by the dashed line. The hyperfine field distribution with fixed smaller isomer shift δ = 0.28 mm/s was attributed to the contribution of magnetite tetrahedral sublattice A or to maghemite [49]. Another hyperfine distribution had larger fixed isomer shift of δ = 0.66 mm/s. The isomer shift of the contribution of magnetite octahedral B sublattice is larger because of the presence of both Fe^2+^ and Fe^3+^. It is noteworthy that using the fixed isomer shift of hyperfine field distribution, the contribution of B sublattice can be separated for magnetite but not for maghemite γ-Fe_2_O_3_ without Fe^2+^. For bulk magnetite, hyperfine field *B*_0_ = 49 T for A and ≈ 46 T for B sublattice [49]. Average hyperfine fields *<B>* of both distributions were 26% lower than those of bulk magnetite, indicating superparamagnetic relaxation of nanoparticles (Table 1). The characteristic size of maghemite/magnetite nanoparticles ≈11.6 nm was obtained applying hyperfine field dependence on the size of nanoparticles *B = B*_0_ (1 − *kT/2 KV)*, where *k* is the Boltzmann constant, *T* is temperature, *K* ≈ 10^4^ J/m^3^ is magnetic anisotropy of magnetite and *V* is average volume of nanoparticles [50]. According to the sub-spectra ratio, there was about 14% Fe^2+^ of all iron. 

**Figure 5:**
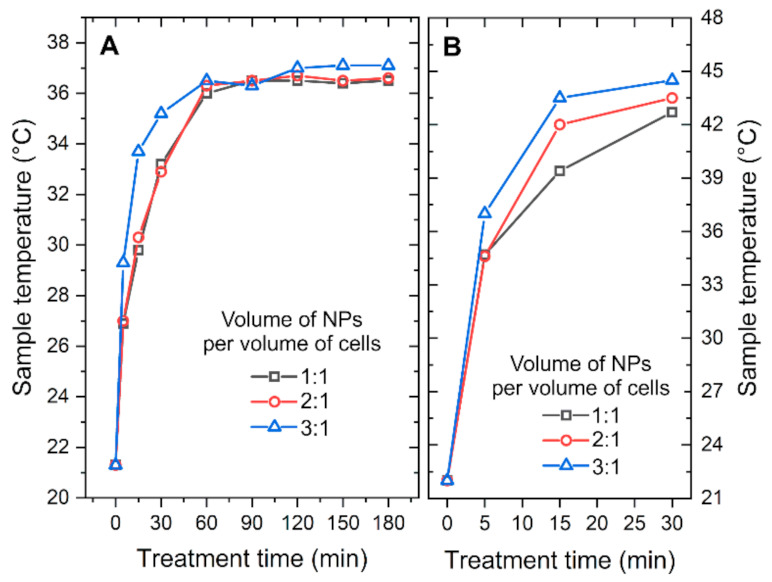
Measured dependence of sample temperature on concentration of nanoparticles and treatment time. (**A**) 100 kHz low magnetic field generator and (**B**) 200 kHz high magnetic field generator.

Considering that the developed NPs have both the biological activity and a thermal physical stressor due to superparamagnetic relaxation, the increase of temperature and the resulting hyperthermia was evaluated for each concentration of NPs. The results are summarized in Figure 5.

As it can be seen in Figure 5A, the low AMFs resulted in a saturated temperature after 60 min, while the dependence on the NP concentration was weak and not statistically significant. The highest temperature of 37 °C was reached. In the case of high AMFs (Figure 5B), the differences were more profound. The temperature reached saturation after 30 min and was almost 45 °C. 

**Figure 6:**
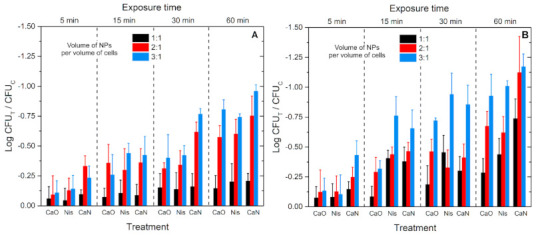
The viability response of *L. innocua* to thermal stress and nisin-loaded (CaN), nisin-free (CaO) magnetic nanoparticles or nisin-only solution (Nis). (**A**) 37 °C experiments; (**B**) 45 °C experiments. In all cases the treatment data (CFU_T_) are normalized to untreated control samples (CFU_C_).

Further, experiments with cells incubated with or without nanoparticles for up to 1 h at different equivalent temperatures were performed. The results are summarized in Figure 6.

As expected, the efficacy of nisin or CaO and CaN nanoparticles depends on concentration and temperature. Nevertheless, cell exposure to 1 h 37 °C thermal stress with CaN NPs potentiates the efficacy of the treatment to reach 1 log inactivation only (Figure 6A). Similar efficacies are more rapidly reached (15–30 min) when 45 °C thermal stress is applied (Figure 6B); however, in all cases the efficacies are below 1.25 log reduction. It should be also noted that in the case of AMF treatments, the NPs heat the samples gradually (refer to Figure 5). However, the data presented in Figure 6 are for stable temperature incubation; thus, more thermal stress is experienced by the cells compared to AMF treatment. Considering the acquired data and weak effects of temperature, further experiments targeting cells in AMFs were performed. 

**Figure 7:**
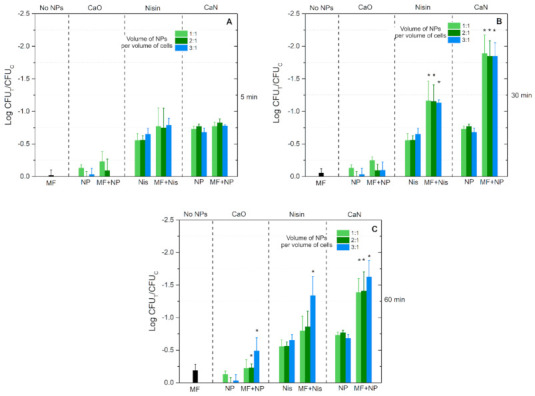
The viability response of *L. innocua* to low alternating magnetic field (10 mT, 100 kHz) and activated nisin-loaded (CaN), nisin-free (CaO) magnetic nanoparticles or nisin-only solution (Nisin). (**A**) After 5 min exposure; (**B**) 30 min exposure and (**C**) 60 min exposure. In all cases the treatment (CFU_T_) is normalized to untreated control samples (CFU_C_). Asterisk (*) corresponds to statistically significant difference (*p* < 0.05) versus NP-only or nisin-only treatment.

In order to preserve a predominantly non-thermal treatment the exposure time was limited to 60 min for the low AMF generator and 30 min for the high AMF generator. The results after low AMF treatment are summarized in Figure 7. 

It can be seen that the exposure time for the MF-only treatment has a weak influence on the cell viability. In the case of 5 and 30 min treatments, the differences are not statistically significant, while for the 60 min protocol (Figure 7C) a minor <0.25 log viability reduction was detected. Exposure of the cells to magnetic nanoparticles only (CaO without heating) did not result in any effect, or it fell within the standard deviation of data independent of the applied concentration of NPs. However, in the case of nisin-loaded magnetic nanoparticles (CaN without heating), a detectable drop in cell viability was observed. On average, the CaN treatment resulted in a better treatment efficacy compared to nisin-only treatment—the highest difference was close to 1 log reduction of cell viability. 

Finally, the combination of the treatments with low alternating magnetic fields potentiated the inactivation efficacy during the 30 and 60 min protocols, while for the 5 min procedure the effect was non-detectable. Similarly to MF-free treatment, the dependence on the NP concentration was not profound in most of cases, indicating a saturated treatment efficacy. It was concluded that application of low AMFs (10 mT, 100 kHz) with the proposed nanoparticles has no practical application in a food processing context due to weak inactivation efficacy. The difference between thermal stress response (Figure 6A) and the best low AMF protocol (Figure 7B) is only 1–1.5 log of cell viability reduction.

**Figure 8:**
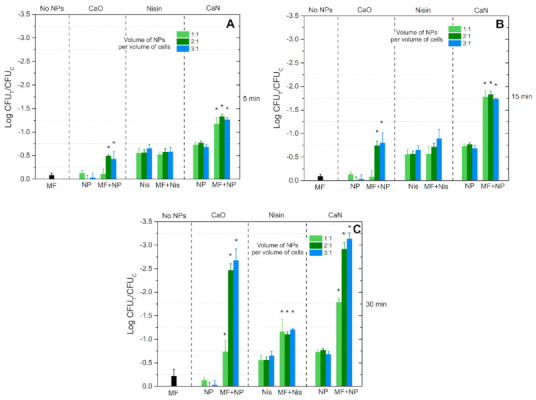
The viability response of *L. innocua* to high alternating magnetic field (125 mT, 200 kHz) and activated nisin-loaded (CaN), nisin-free (CaO) magnetic nanoparticles or nisin-only solution (Nisin). (**A**) After 5 min exposure; (**B**) 15 min exposure and (**C**) 30 min exposure. In all cases the treatment (CFU_T_) is normalized to untreated control samples (CFU_C_). Asterisk (*) corresponds to statistically significant difference (*p* < 0.05) versus NP-only or nisin-only treatment.

Similar analysis was performed for the high AMF (125 mT, 200 kHz) treatment. The results are summarized in Figure 8. 

Similarly to the low AMF treatment, the MF-only (125 mT, 200 kHz) procedure resulted in a minor decrease of the cell viability (independent of the exposure time). Nevertheless, in the case of high AMFs, the 5 min protocol (Figure 8A) triggered a statistically significant difference in the inactivation rate when CaO nisin-free NPs were used, contrary to the 10 mT treatment. 

An exposure time-dependent response was acquired with the high AMF treatment, i.e., the inactivation efficacy gradually increased, and the highest inactivation of 3 log reduction was observed (Figure 8C), which is a significant result. If compared to the response mediated by temperature (Figure 6B), additional 2 log reduction was associated with the AMF component. 

**Figure 9:**
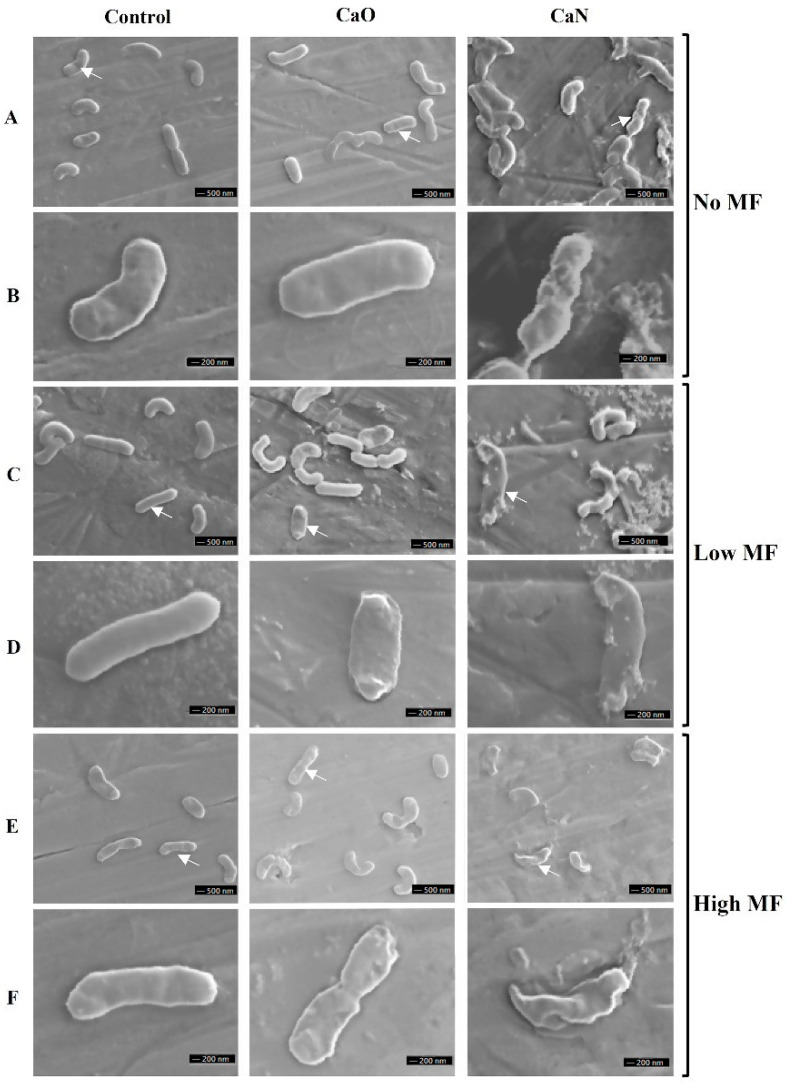
Representative scanning electron microscopy images of various protocols with and without nanoparticles. (**A**) Untreated and samples with CaO and CaN only; (**C**) 10 mT, 100 kHz/60 min treated and samples after CaO and CaN + 10 mT AMF procedure; (**E**) 125 mT, 200 kHz/30 min treated and samples after CaO and CaN + 125 mT AMF procedure; magnified images of bacteria highlighted with white arrows are shown in (**B**,**D**,**F**).

The SEM analysis of the applied protocols was further introduced in the study. The representative exemplary images are shown in Figure 9. 

It can be seen that untreated bacteria and cells after MF-only treatment morphologically look the same. However, when CaO is applied with MF treatment, differences can be seen (i.e., Figure 9F, CaO after 125 mT treatment)—the rigid structure of the cell is ruined, and the integrity of the membrane is questionable. A similar result is observed when CaN-NP-only treatment is used (Figure 9A,B, CaN). Lastly, when the nisin magnetic NPs are combined with AMF treatment, the cellular damage is irreversible, and the observed effects (Figure 9F, CaN) are in good agreement with the viability data (Figure 8C). To summarize, the SEM images adequately represent the viability data overviewed in Figure 7 and Figure 8. 

## 4. Discussion

It was shown that the application of alternating magnetic fields with functionalized magnetic nanoparticles can effectively manipulate the initial resistance of bacteria to nisin. Due to the pathogenicity of the environmentally persistent *L. monocytogenes*, it is challenging to safely conduct large-scale experiments using this bacteria. However, nonpathogenic *L. innocua*, widespread in the environment and in food bacteria, have similar genotypic and phenotypic characteristics to *L. monocytogenes* [51]; thus, this species was employed as a surrogate bacteria giving a safety margin to protect researchers and preventing exposure to the pathogens.

We showed that 10 and 125 mT AMFs (without NPs) have no-to-weak effect on the viability of the *L. Innocua*. When combined with magnetic nanoparticles the inactivation efficacy improved significantly. The observed effects could be partly attributed to the thermal stress and mild thermal treatment [52,53], which is induced due to the relaxation losses in magnetic nanoparticles. The capability to potentiate the effects of nisin nanoparticles by mild thermal treatment was reported previously [37]. Nevertheless, the effects of thermal stress are limited to 1 log reduction of bacterial cell viability, which is insignificant in a food processing context. Application of 125 mT AMFs allowed up to 3 log reduction of cell viability to be reached.

Alternating magnetic fields of similar parameters (100–200 mT, kHz range) were reported to trigger inactivation of *E. coli* when longer exposure times (6–16 h) were used [54]. Such exposure rates limit the economic benefits and applicability of the methodology in industry. However, in our case the treatment was limited to 30 min, featuring a contactless and predominantly non-thermal methodology. Nevertheless, there are some reports on *Saccharomyces cerevisiae* yeast treated by a 5 mT magnetic field for 30 min that report inactivation in the 30% to 70% range [55], while others report growth stimulation [56]. It was also shown that magnetic fields in the mT range can affect enzyme activity and lipid peroxidation [57]. Our data indicate that there is a tendency of viability reduction after magnetic field treatment without nanoparticles. However, descriptive conclusions on the biophysics of the phenomena cannot be formed due to weak and mainly statistically insignificant effects. 

When combined with functionalized NPs, the 10 mT treatment returned insignificant results (industry-wise) independent of exposure time and nanoparticles used since the induced inactivation was too low. Surprisingly, the 30 min treatment resulted in a better treatment efficacy compared to the 60 min treatment. We believe that the result could be attributed to an increased bacteria growth in higher temperature (37 °C). Such a phenomenon was reported previously [58,59].

The major improvements of the AMF methodology in our work lie in the potentiation of bioactive effects of functionalized and nisin-free ferromagnetic nanoparticles in the 125 mT magnetic field. Of course, partly, the inactivation efficacy was affected by mild temperature increase according to magnetic hyperthermia methodology [60]; however, it was not the dominant stressor. The non-thermal effect of NPs could be attributed to local field amplification due to conductive nanoparticles in close proximity with the cell membrane [43]. Combined with relaxation losses and resulting mild thermal treatment, the fluidity of the cell membrane could also have been altered [61]. Nevertheless, the proposed methodology offers precise control of the treatment parameters. In terms of temperature and considering the physics behind this phenomena, a further increase of the AMF frequency is required to produce more hysteresis losses [62] and thus more effective local heating and nisin activity improvement. Differently from microwave heating processing steps where the heat is generated within the food itself [63], functionalized magnetic nanoparticles enable activation of the nanoparticle itself in close proximity to target microorganisms, followed by heat diffusion in the whole volume. This may potentially allow mild thermal treatments (<45 °C) of the food with nisin exposed to higher temperature (due to magnetic NP functionalization) where it can act more rapidly in the optimal bactericidal mode [64]. Further multiparametric analysis is required including optimization of treatment time, NP concentration and increase of magnetic field frequency. Considering the effects of AMFs, further increase of the pulse amplitude is required, which according to our data may further improve the inactivation of microorganisms. A shorter period of pulses may also positively affect the treatment outcome due to the *dB/dt*-dependent induced electric field component [65].

## 5. Conclusions

To conclude, functionalized magnetic nanoparticles have high actuality in drug delivery systems [66], the cancer treatment context [67] or in the detection of bacterial pathogens [68]. Superparamagnetic nanoparticles do not have remnant magnetization in the absence of the external magnetic field and are considered to have no toxicity [69]. Moreover, iron oxide is already approved by FDA for medical and food applications, making nanoparticles good candidates for use in food pathogen biocontrol [70]. Our data indicate that there is a niche in mild hyperthermia-assisted food processing steps that can be utilized to overcome resistance to food-compatible bacteriocins and potentiate the inactivation effects of AMFs several-fold. The methodology is a competitive contactless alternative to currently established PEF-mediated treatments.

## Figures and Tables

**Table 1:** Parameters of fitting to Mössbauer spectrum: isomer shift δ, quadrupole shift *2ε*, average hyperfine field *<B>* of distribution and relative area *A* attributed to magnetite ^§^.

Sub-spectrum	*δ*, mm/s	2*ε*, mm/s	*<B>, T*	*A*, %
Magnetite A (Fe^3+^) or maghemite	0.28 *	0.02 ± 0.01	36.2	69
Magnetite B (Fe^2+^ + Fe^3+^)	0.66 *	−0.23 ± 0.03	33.2	27
Superparamagnetic singlet	0.43 ± 0.04	-	-	4

^§^ Isomer shift is given with respect to α-Fe. * fixed.

## Data Availability

Data available from the corresponding author V.N. on request.
